# Geldanamycin, a Naturally Occurring Inhibitor of Hsp90 and a Lead Compound for Medicinal Chemistry

**DOI:** 10.1021/acs.jmedchem.4c01048

**Published:** 2024-10-03

**Authors:** Russell R. A. Kitson, Dominika Kitsonová, David Siegel, David Ross, Christopher J. Moody

**Affiliations:** † Department of Organic and Bioorganic Chemistry, Charles University, Faculty of Pharmacy in Hradec Králové, Akademika Heyrovského 1203, 50005 Hradec Králové, Czech Republic; ‡ Datwyler Sealing Technologies CZ Ltd., Polní 224, 50401 Nový Bydžov, Czech Republic; § Department of Pharmaceutical Sciences, University of Colorado Anschutz Medical Campus, 12850 East Montview Boulevard, Aurora, Colorado 80045, United States; ∥ School of Chemistry, University of Nottingham, University Park, Nottingham NG7 2RD, U.K.

## Abstract

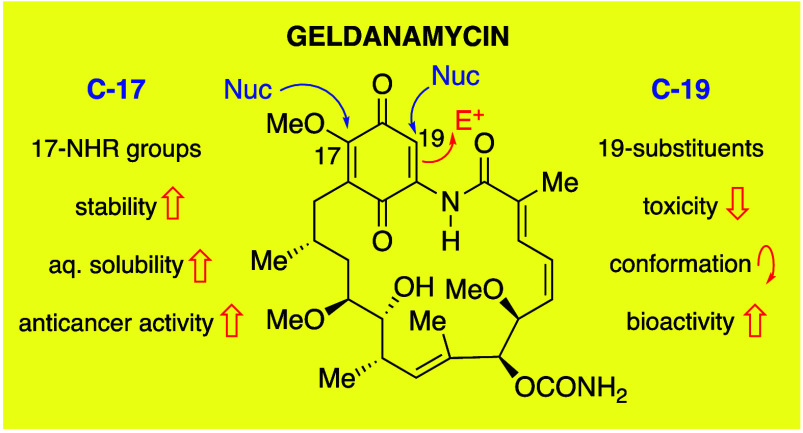

Geldanamycin remains a driver in the medicinal chemistry of heat shock protein 90 (Hsp90) inhibition, even half a century after its original isolation from nature. This Perspective focuses on the properties of the benzoquinone ring of the natural product that enable a range of functionalization reactions to take place. Therefore, inherent reactivity at C-17, where the methoxy group serves as a vinylogous ester, and at C-19 that demonstrates nucleophilic, enamide-type character toward electrophiles, and also as a conjugate acceptor to react with nucleophiles, has facilitated the synthesis of semisynthetic derivatives. Thus, a range of C-17-substituted amine derivatives has been investigated in oncology applications, with a number of compounds in this series reaching clinical trials. In contrast, the 19-position of geldanamycin has received less attention, although 19-substituted derivatives offer promise with markedly reduced toxicity compared to geldanamycin itself, while retaining Hsp90 inhibitory activity albeit with diminished potency in cellular studies.

## Significance

- Heat shock protein 90 (Hsp90) remains an attractive target for drug discovery with potential applications in oncology, HIV/AIDS, malaria and neurodegenerative disease.
- Early work was influenced by a natural product, geldanamycin, a benzoquinone ansamycin, that continues to provide inspiration for drug discovery programs.
- This Perspective highlights the role of geldanamycin as a versatile starting point for medicinal chemistry, with the potential to create a wide range of semisynthetic derivatives by modification of the quinone ring.

## 1 Introduction

Since the dawn of the subject, organic chemists have been intrigued by the complex and fascinating structures of molecules obtained from nature. As a result of biosynthetic pathways, naturally occurring compounds have the prerequisites for binding to proteins and penetrating cell membranes, and are therefore often medicinally active.^1^ One such natural product that has attracted considerable attention for over half a century is the benzoquinone ansamycin (BQA) geldanamycin.

**Figure 1:**
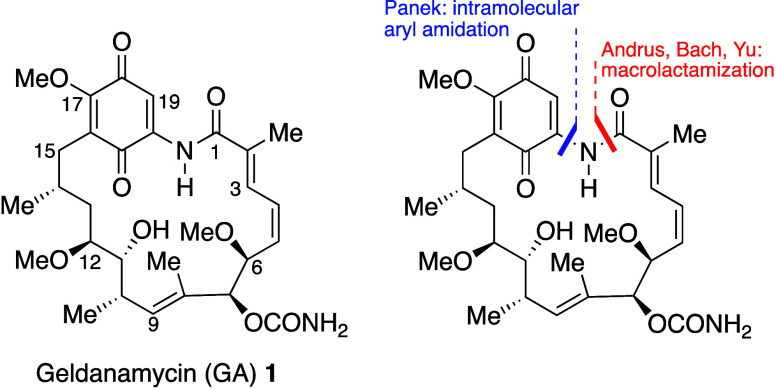
Structure of geldanamycin **1**, showing ring numbering system, and macrocyclization steps in the total syntheses.

Geldanamycin (GA), a yellow colored compound first isolated from *Streptomyces hygroscopicus* var. *geldanus* in 1970 was found to possess antibacterial properties.^2^ The structure **1** (Figure 1), the first ansamycin to contain a benzoquinone rather than the more usual naphthoquinone, as, for example, in the rifamycins, was assigned on the basis of extensive spectroscopic studies by Rinehart and co-workers.^3^ Like other members of the ansamycin family, geldanamycin is biosynthesized by the polyketide pathway, with the shikimate-derived 3-amino-5-hydroxybenzoic acid serving as the starter unit for polyketide synthases. The chain is extended using malonyl-CoA or methylmalonyl-CoA units, followed by postpolyketide modifications.^4,5^

As a complex molecular structure with a 19-membered macrocyclic ring encompassing six stereocenters and three alkenes, geldanamycin has unsurprisingly attracted the attention of synthetic organic chemists, and to date has been the subject of three total syntheses from the groups of Andrus (2002),^6^ Panek (2008)^7^ and Yu (2021).^8^ Another synthesis by Bach and co-workers (2012) was thwarted at the final deprotection step.^9^ Although details of these syntheses are beyond the scope of this Perspective, it is interesting to note that Andrus, Bach and Yu employed a conventional amide formation reaction to close the 19-membered macrolactam, whereas Panek used a copper(I) catalyzed intramolecular aryl amidation reaction (Figure 1).^10^

Geldanamycin was initially reported to possess antibacterial properties,^2^ and following his seminal studies on the structure of the antibiotic, Rinehart subsequently investigated GA as an inhibitor of various RNA-dependent DNA polymerases.^11,12^ However, it was Neckers’ 1994 discovery of GA’s potent and specific inhibition of the molecular chaperone, heat shock protein 90 (Hsp90),^13^ that triggered a huge increase in interest in this natural product.^14^ As noted in a previous article,^10^ the annual number of Hsp90- and geldanamycin-related publications rose from *ca*. 200 in 1994 to *ca*. 950 in 2011, and by 2021 had reached in excess of 1200.

Hsp90, one of the most abundant proteins in eukaryotic cells, is a 90-kDa heat shock protein that is an ATP-hydrolysis driven molecular chaperone that functions to promote the conformational stabilization and activation of a wide range of client proteins.^15−17^ There are hundreds of Hsp90 interacting proteins including client proteins, cochaperones and other proteins (Hsp90 interactors, accessed 04/2024, https://www.picard.ch/downloads/Hsp90interactors.pdf). In cancer cells, Hsp90 client proteins include oncoproteins, transcription factors, kinases and additional proteins important for growth, survival and drug resistance,^15,18^ and by targeting Hsp90 it is possible to block multiple oncogenic pathways.^19^ Inhibition of Hsp90 also leads to release of the transcription factor heat shock factor 1 (Hsf1) resulting in the induction of additional protective heat shock proteins including Hsp70 and Hsp27 which may assist in the appropriate folding of proteins or promote proteasomal degradation of misfolded proteins.^20−22^ Depletion of Hsp90 client proteins and induction of other protective heat shock proteins are commonly used in *in vitro* and *in vi*vo studies as biomarkers of Hsp90 inhibition.^23,24^ Hsp90 inhibitors may also induce protective autophagy which may facilitate the degradation of protein aggregates.^25^ The multiple effects of Hsp90 inhibition has led to the consideration of Hsp90 as a druggable target in a range of diseases other than cancer, including neurodegenerative diseases, malaria and HIV/AIDS.^21,26−28^ As a result, Hsp90 has emerged as one of the most attractive and widely studied molecular targets for small molecule inhibition, particularly in oncology, with pimitespib (Jeselhy), a nitrogen heterocycle (MW = 454), being the first Hsp90 inhibitor approved for clinical use in 2022.^29,30^ At present, the approval is limited to Japan for use against gastrointestinal stromal tumors, although the compound is still undergoing clinical trials in the US and EU.

As discussed, in cancer cells, Hsp90 can serve to prevent the misfolding or degradation of numerous overexpressed or mutated oncoproteins, and as a result, many cancers increasingly rely upon Hsp90 for survival.^15,23,31^ Hsp90 derived from tumor cells was reported to have a 100-fold higher binding affinity for the Hsp90 inhibitor 17-AAG **2a** (see below) than Hsp90 from normal cells due to the presence of multichaperone complexes with high ATPase activity, offering a considerable window for anticancer drug targeting.^32^ Therefore, medicinal chemists have developed a range of novel targeted agents that might effectively treat numerous cancer types,^33^ and although GA itself has not progressed to the clinic due to unacceptable liver toxicity,^34^ it has proven to be an excellent lead compound for drug discovery.

**Figure 2:**
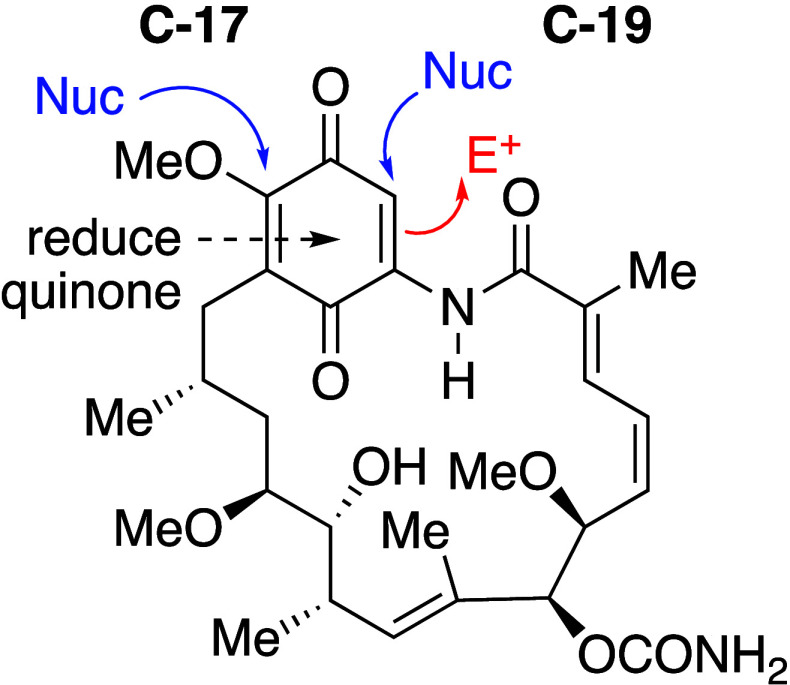
Reactivity of the quinone ring of geldanamycin.

The versatility of GA as a starting point for medicinal chemistry lies in the fact that it is accessible in reasonable quantities via fermentation (5 g = *ca*. $1000, March 2024), and also in the unique properties of the benzoquinone ring as outlined in Figure 2. The potential for reactions at C-17 and C-19 of GA to create semisynthetic derivatives of the natural product was recognized by Rinehart,^11,12^ following on from his seminal work on the structure determination.^3^ Subsequently, a range of semisynthetic derivatives became readily available, the majority of which derive from modification at the C-17 position. The C-17 methoxy group present in the natural product is equivalent to a vinylogous ester, and readily undergoes addition–elimination reactions with a range of nucleophiles including hydroxide,^12^ alkoxides,^35^ and, in particular, with amines, although bulky amines preferentially attack at C-19 (see later).^36^ In contrast, the 19-position of geldanamycin has received much less attention. However, as described below, the reactivity of the natural product at C-19 can be exploited in two ways; either by realizing the nucleophilic, enamide-type character of the amino-quinone moiety to react with electrophiles such as iodine, or the ability of the quinone to act as a conjugate acceptor, reacting with nucleophiles such as thiols, amines and alcohols.

There are also the reactions of the benzoquinone ring itself to consider. As discussed below, attack at C-17 or C-19 with “double” nucleophiles such as diamines can result in subsequent cyclization onto the C-18 quinone carbonyl. However, the behavior of quinones is dominated by their inherent electrophilic reactivity and their reduction both under chemical and biological conditions. Indeed, the cytotoxicity of quinones is often associated with their ability to alkylate biological nucleophiles such as thiols, and by redox cycling that can lead to oxidative stress due to the formation of reactive oxygen species. It has been shown that cytotoxicity of the BQAs such as GA increases as their respective reduction potential increases.^37^ Hence introduction of an electron-releasing substituent by substitution of an amine at C-17, decreases the reduction potential, and in general decreases the toxicity.

By focusing on the reactivity of its benzoquinone ring rather than the ansa-chain, this Perspective aims to highlight the role of GA as a lead compound for medicinal chemistry, and to emphasize that natural products continue to provide inspiration for our efforts to discover new medicines.

## 2 17-Substituted Derivatives of Geldanamycin

### 2.1 Amino-Substituted Analogues and Their Clinical Development

**Scheme 1:**
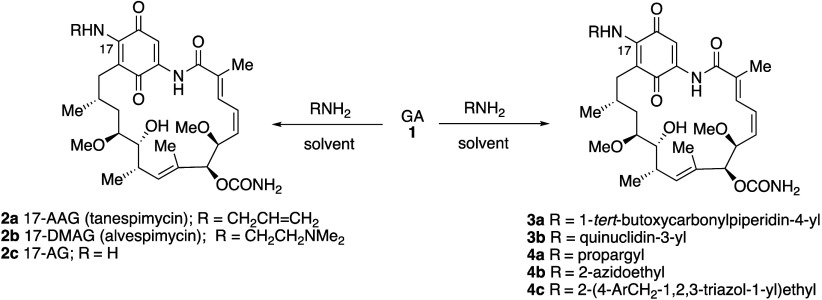
Nucleophilic Addition of Amines at C-17, Followed by Elimination of the Methoxy Group, Yields a Wide Range of 17-Amino-17-demethoxygeldanamycin Derivatives, Including Compounds **2a** and **2b**, That Have Been Clinically Evaluated, Compounds **3**, Containing an Amino Substituent for Further Elaboration, and Compounds **4**, Involved in Click Chemistry

**Figure 3:**
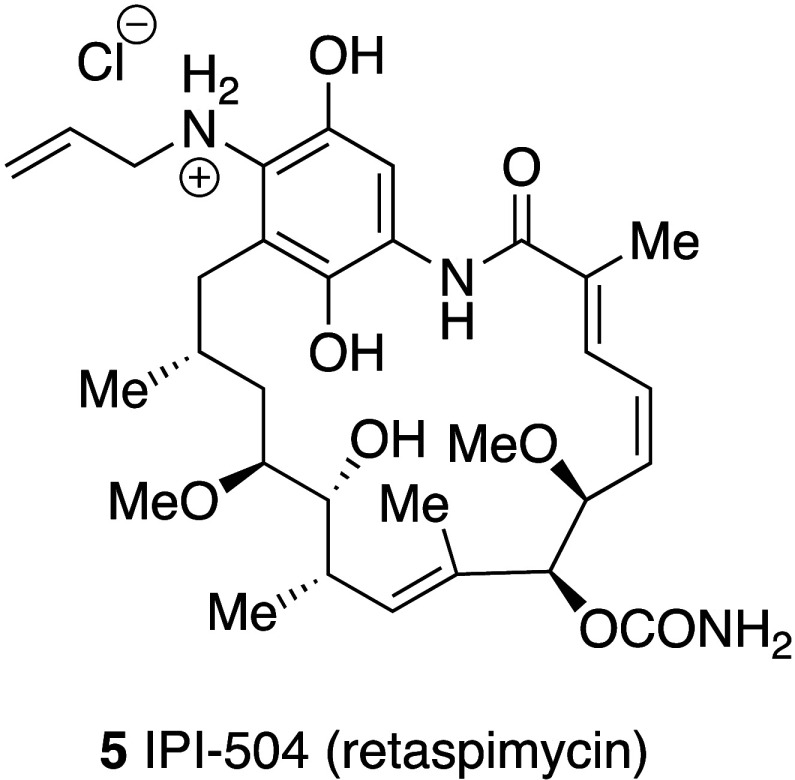
Structure of 17-AAG hydroquinone (IPI-504, retaspimycin).

Despite GA **1** providing a lead for drug discovery, it did not progress to the clinic, due to poor solubility and stability and, in particular, unacceptable liver toxicity.^34^ Nevertheless, a multitude of semisynthetic compounds formed by nucleophilic addition–elimination of amines at C-17 have been widely studied.^36,38^ Such compounds are readily generated by treatment of GA with a primary or secondary amine in a suitable solvent such as chloroform or DMF to give the corresponding 17-amino derivatives as purple solids (Scheme 1). Examples include the more stable derivatives 17-allylamino-17-demethoxygeldanamycin (17-AAG, tanespimycin) **2a**, 17-*N*,*N*-dimethylaminoethylamino-17-demethoxygeldanamycin (17-DMAG, alvespimycin) **2b**, and 17-amino-17-demethoxygeldanamycin (17-AG) **2c**,^36^ a compound that also occurs naturally.^39^ In addition to being more potent than 17-AAG in melanoma models, 17-DMAG has the advantage of being considerably more water-soluble.^40^ Similarly, 17-AAG hydroquinone, as its hydrochloride salt **5** (IPI-504, retaspimycin) (Figure 3) is also more water-soluble.

The ATP binding site in the *N*-terminal domain of Hsp90 is the target of the majority of Hsp90 inhibitors including the BQAs.^10,33^ The prototype Hsp90 inhibitor was geldanamycin, but as discussed above, it did not move forward to clinical development due to off-target toxicities and poor solubility.^34^ Second generation benzoquinone ansamycin derivatives formed by C-17 substitution included 17-AAG, **2a**, 17-DMAG, **2b**, and 17-AG **2c (**Scheme 1). 17-AAG is poorly soluble leading to formulation and delivery problems while both 17-AAG and 17-DMAG demonstrated significant toxicities in Phase I and II trials including hepatotoxicity.^41−44^

An attractive approach was to create a more water-soluble analogue of 17-AAG by reducing the quinone moiety to the hydroquinone, IPI-504 (retaspimycin hydrochloride). IPI-504 and 17-AAG were proposed to exist in a redox equilibrium both *in vitro* and *in vivo*.^45^ IPI-504 was well tolerated and demonstrated modest single agent activity in a Phase I study of relapsed or refractory multiple myeloma,^46^ and some evidence of antitumor activity in gastrointestinal stromal tumors.^47^ Minimal or modest clinical activity and mild to unacceptable toxicities dependent on dosage and schedule were observed in Phase II clinical trials of IPI-504 in castration resistant prostate cancer,^48^ nonsmall cell lung cancer,^49^ and in combination trials with trastuzumab in metastatic HER2-positive breast cancer.^50^ A useful review and summary of the development and clinical activity of Hsp90 inhibitors including IPI-504 **5** (Figure 3) in the treatment of nonsmall cell lung cancer has been published.^51^ Despite initial promise, IPI-504 is no longer in clinical development as an anticancer agent.

Although IPI-504 remains the only geldanamycin *hydroquinone* derivative to be extensively investigated, the importance of quinone reduction should not be underestimated. Mechanistically, our work showed that reduction of the quinone functionality to the hydroquinone in a series of BQAs led to superior Hsp90 inhibition due to increased H-bonding and more favorable binding of the hydroquinone in the ATP binding site of Hsp90.^52,53^ Biologically, an efficient method of reduction to generate the active hydroquinone forms of BQA Hsp90 inhibitors was via reduction of the quinone by the obligate two electron reductase NAD(P)H:quinone oxidoreductase 1 (NQO1, QR1). NQO1 is expressed at high levels in many human solid tumors,^54,55^ providing an efficient mechanism for generating the more active hydroquinone form *in situ* in tumor cells. Pharmacological inhibition of NQO1 led to decreased BQA-induced Hsp90 inhibition and toxicity to human cancer cells whereas genetic overexpression of NQO1 led to increased BQA-induced toxicity.^52^ The role of NQO1 in the bioreduction of quinone-containing antitumor agents such as mitomycin C,^56^ aziridinylbenzoquinones including RH1,^57^ β-lapachone^58^ and streptonigrin^59^ has been well characterized.^60^

Persistent NRF2 upregulation has been documented in many tumors due to activating mutations in the Keap1-NRF2 pathway resulting in overexpression of many genes including NQO1.^61^ Such tumors are often resistant to standard therapy resulting in poor patient outcomes.^61^ Using cell lines, screens have been developed for compounds that exert synthetic lethality with NRF2 overexpression.^62^ Quinone-based Hsp90 inhibitors^63^ and mitomycin C^64^ have been identified in such screens and genetic manipulation confirmed that NQO1 was the major NRF2 target gene responsible for bioactivation of 17-AAG, 17-DMAG and IPI-504.^63^

In humans, there are four isoforms of Hsp90: Hsp90α and Hsp90β, found in the cytosol, GRP94 (glucose-regulated protein 94), located in the endoplasmic reticulum, and the mitochondria-localized TRAP-1 (TNF receptor associated protein 1),^65^ GA and related compounds bind to the *N*-terminal domain of Hsp90^66,67^^68^ and it is generally assumed that BQAs such as GA and 17-AAG are pan inhibitors with little selectivity between the four isoforms.^69−71^ The development of isoform-selective inhibitors of Hsp90 has been a recent focus as a potential approach to reduce toxicity and limit the heat shock response considered a detriment in anticancer therapy using Hsp90 inhibitors.^65,70^ Selective inhibition of cytosolic Hsp90α and Hsp90β is particularly challenging due to their structural similarity and overlapping functions but progress has been made toward creating isoform-specific inhibitors.^72−74^

**Scheme 2:**
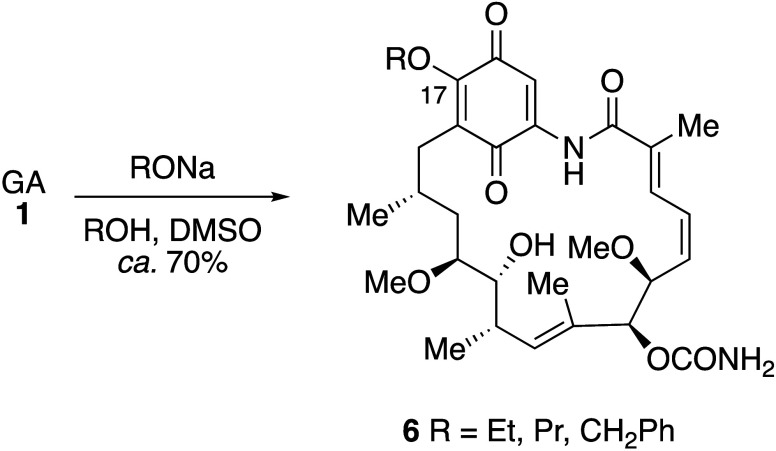
Nucleophilic Addition–Elimination Reactions of Alkoxides at C-17: Synthesis of 17-Alkoxy-17-demethoxygeldanamycin Derivatives

A number of novel 17-amino derivatives have been prepared and evaluated recently. For example, the compound (**3a**, Scheme 1, R = 1-*tert*-butoxycarbonylpiperidin-4-yl) derived by reaction of GA with 4-amino-1-*tert*-butoxycarbonyl-piperidine proved more potent *in vitro* than GA in a panel of five cancer cell lines, more selective compared to normal human dermal fibroblasts (∼30-fold vs. ∼ 2.5-fold), and also more potent than 17-AAG against SKOV-3 (0.083 μM vs. 0.22 μM) and A-549 (0.077 μM vs. > 10 μM) cell lines.^75^ In further studies, the same authors reported that quaternization of 17-amino substituents containing a tertiary amine both reduced toxicity in normal CCD39Lu cells and increased water solubility compared with GA itself. Thus, for example, reaction of GA with 3-aminoquinuclidine gave **3b** (Scheme 1, R = quinuclidin-3-yl) followed by quaternization of the quinuclidine nitrogen with alkyl bromides gave a range of novel analogues for biological evaluation.^76^ Click chemistry has also been used to modify GA at C-17. Thus, treatment of GA with propargylamine yielded a compound (**4a**, Scheme 1, R = propargyl) suitable for further functionalization by reaction with azides. Likewise, reaction of GA with 2-azidoethylamine gave an intermediate (**4b**, Scheme 2, R = 2-azidoethyl) for reaction with terminal alkynes in an alternative click reaction. Hence a wide range of 1,2,3-triazoles was obtained for biological evaluation, with some compounds (**4c**, Scheme 1, R = 2-(4-ArCH_2_-1,2,3-triazol-1-yl)ethyl) having higher anticancer activity than GA (IC_50_ = 0.23–0.41 μM vs. 0.58–0.64 μM for GA) in SkBr-3, SKOV-3 and PC-3 cell lines, with comparable cytotoxicity in healthy cells.^77^ Click chemistry was also used to link the GA scaffold to colchiceine to give colchicine-geldanamycin hybrid compounds that had decreased anticancer activity compared with colchicine itself, but improved activity vs. GA.^78^

### 2.2 O- and S-Substituted Analogues

As discussed below, inhibition of Hsp90 is thought to result in a neuroprotective effect, and this prompted an investigation of a series of 17-*N*-alkyl and *O*-alkyl derivatives of GA.^35^ The *N*-alkyl compounds were prepared by reaction of GA with primary amines in DMF as described above (Scheme 1), whereas the *O*-alkyl derivatives **6** were formed by treatment of GA with 1.4 equivalents of the corresponding sodium alkoxide (Scheme 2). Whereas all of the *N*-alkyl derivatives tested showed some neurotoxicity in P19-derived neurons from a specific mouse embryonal carcinoma cell line, the *O*-alkyl compounds **6** were neuroprotective, although this discovery does not appear to have been followed up. In separate experiments, it was also shown that compounds with larger alkyl substituents such as **6** (R = CH_2_Ph) (IC_50_ = 0.5 μM) and the 17,19-bismethoxy adduct (IC_50_ = >10 μM) were less cytotoxic than GA itself (IC_50_ = 0.1 μM). In contrast to the number of semisynthetic GA derivatives with nitrogen or oxygen substituents at C-17, the sulfur analogues seem to be rare, although the *S*-allyl derivative appears in a patent for antiviral compounds.^79^

### 2.3 C-Substituted Analogues

**Scheme 3:**
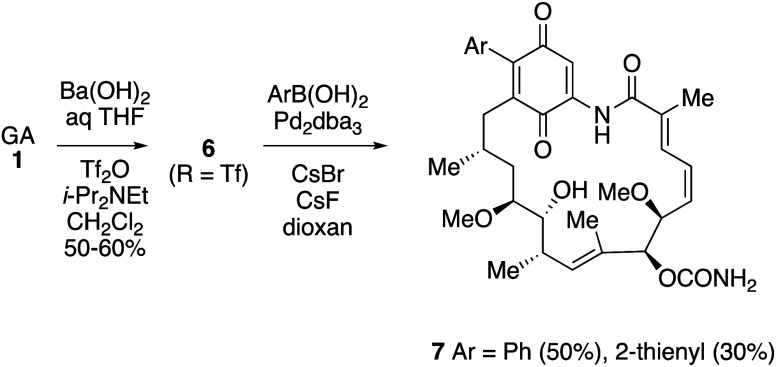
Synthesis of 17-Aryl-17-demethoxygeldanamycin Derivatives

In order to extend the range of C-17 substitutions beyond heteroatom nucleophiles, the introduction of C-nucleophiles using a Suzuki reaction was investigated. Thus, barium hydroxide-mediated hydrolysis of GA, followed by conversion into the corresponding triflate **6** (R = Tf) and reaction with arylboronic acids under palladium(0) catalysis gave the 17-aryl derivatives **7** (Scheme 3).^80^ Only two aryl derivatives **7** (Ar = Ph, 2-thienyl) were prepared, with no attempts reported to extend the palladium-coupling reactions to other groups. Both the 17-phenyl and 17-(2-thienyl) compounds **7** retained good activity against Hsp90 (IC_50_ = 3 and 1.7 μM, respectively), although they performed less well in other assays, for example an approximate 2-fold decrease in HER2 degradation vs. the 17-aminoquinones tested, and were not progressed.

Interestingly, another C-17-substituted analogue of GA was found to occur naturally. The 17-hydroxymethyl compound was isolated from another *S*. *hygroscopicus* strain, although it showed reduced cytotoxicity (>80-fold compared to GA itself) against SkBr3 cells.^81^

### 2.4 C17–C18 Cyclic Analogues

**Scheme 4:**
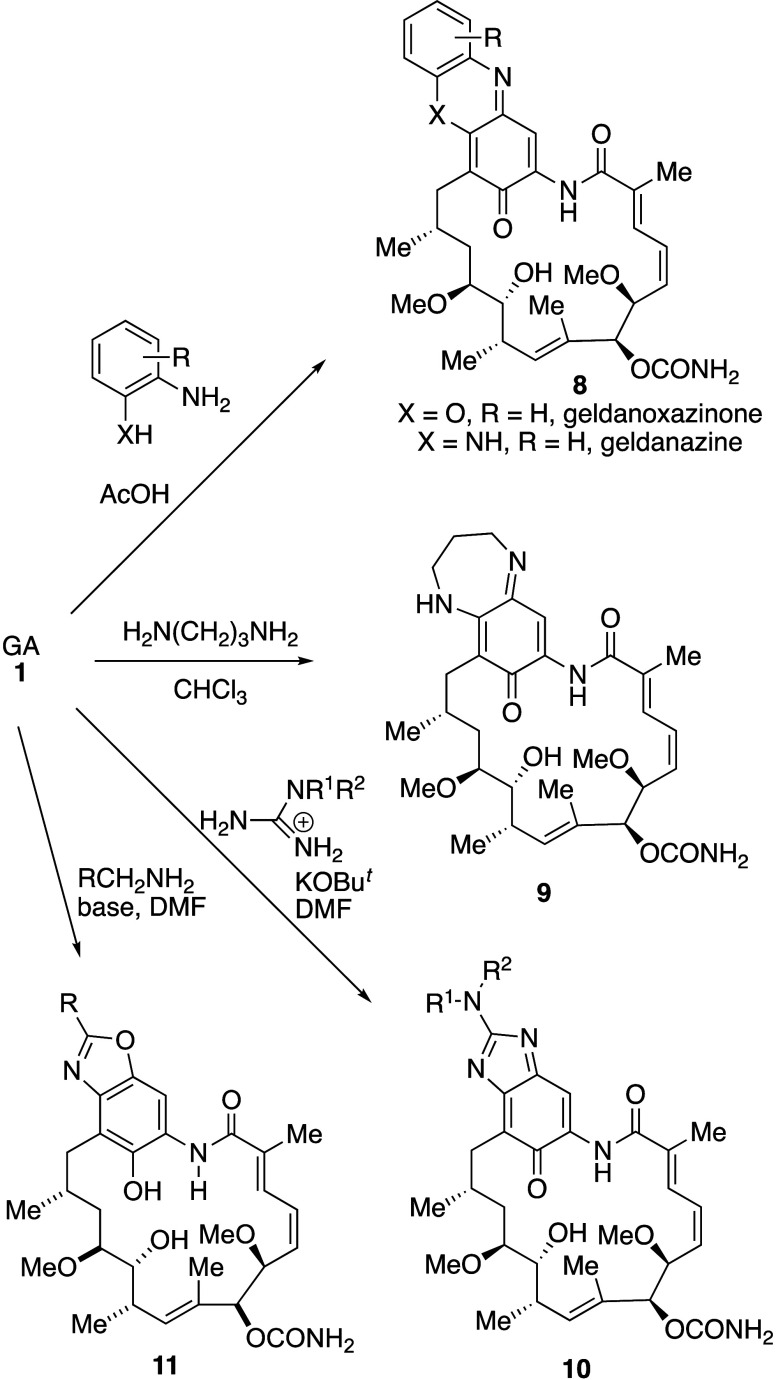
Nucleophilic Addition of Amine or Phenols at C-17, Followed by Elimination of the Methoxy Group and Cyclization to C-18

In his early work, Rinehart showed that a new class of GA derivatives could be prepared by reaction with double nucleophiles, whereby initial reaction at C-17 was followed by cyclization onto the C-18 quinone carbonyl group.^12^ Thus, hydrolysis of GA, followed by reaction with 1,2-phenylenediamines or 2-aminophenols gave the phenazines **8** (X = N) and phenoxazines **8** (X = O), named geldanazines and geldanoxazinones, respectively (Scheme 4). Although the geldanazines and geldanoxazinones were originally proposed as inhibitors of bacterial DNA-dependent RNA polymerases, they had poor activity. On the contrary, with the exception of the geldanazine itself **8** (X = NH, R = H) the compounds were highly effective inhibitors of tumor virus DNA polymerase. Thus, RNA-dependent DNA polymerase (RDDP) from Rauscher leukemia virus (RLV) was inhibited to the extent of 51–96% compared with only 4–19% with GA itself. The unsubstituted compounds **8** (R = H) were also investigated.^36^ Whereas, in studies using SkBr-3 cells, geldanoxazinone **8** (X = O, R = H) was *ca*. 6-fold less potent than GA in depleting the Hsp90 client erbB-2 (p185, HER2), geldanazine **8** (X = NH, R = H) was essentially inactive. The reaction of GA with other double nucleophiles was also investigated, with 1,3-diaminopropane giving the diazepane derivative **9** in low yield and a series of guanadinium salts giving the deep-green, fused imidazoles **10** (Scheme 4). While diazepane **9** was *ca*. 4-fold less active than GA in depleting erbB-2 in SkBr3 cells, the imidazole analogue **10** (R^1^ = R^2^ = Me) was slightly more potent than GA in the same assay.^36^

In a recent development of the addition–elimination reactions of amines at C-17 of geldanamycin (Scheme 1), addition of amines RCH_2_NH_2_ followed by treatment with a base such as tetramethylguanidine resulted in cyclization to give the benzoxazole hydroquinone derivatives **11** (Scheme 4).^82^ The proposed mechanism involves two tautomeric hydrogen shifts of the initial “normal” C-17 amine adduct, followed by cyclization of the C-18 oxygen onto an exocyclic imine, albeit via a disfavored 5-*endo-trig* process, with subsequent aerial oxidation. In breast, ovarian and prostate cancer cell lines (SkBr-3, SKOV-3 and PC-3), the nonquinone benzoxazoles **11** generally showed comparable anticancer activity (IC_50_ = 0.71–0.99 μM) to GA (IC_50_ = 0.58–0.64 μM, but with greater selectivity over normal human dermal fibroblasts.

Despite the volume of work directed at the modification of the GA nucleus at C-17, particularly in the development of the amino derivatives discussed in Section 2.1, and the promising biological activity in a range of preclinical assays, clinical trials with such C-17 amino derivatives have been unsuccessful due to unacceptable toxicities.

## 3 19-Substituted Derivatives of Geldanamycin

In contrast to the 17-position, reactions at the 19-position of GA have received much less attention from medicinal chemists. This is surprising since the reactivity of the natural product at this position can be exploited in two ways as outlined in Figure 2, either by taking advantage of the nucleophilic, enamide-type character of the amino-quinone, or the ability of the quinone to act as a conjugate acceptor, and both these modes of reactivity were recognized early in the history of GA. Thus, Rinehart showed that GA would undergo a Mannich reaction at C-19 to give an imine, subsequently converted into the hydrazones **29** (X = NR_2_) and oximes **29** (X = OR) described below (see Scheme 9).^11^ Somewhat later it was shown that GA underwent remarkably selective bromination or iodination at C-19.^83^ In terms of conjugate addition to C-19, whereas most amine nucleophiles attack GA at C-17 as discussed above, bulky amines attack at C-19.^36^

The C-19 position of GA is also important for other reasons. The clinical development of GA was halted due to unacceptable liver toxicity, and the authors speculated that this could be a result of hepatic metabolism possibly involving addition of glutathione at C-19.^34^ A decade later it was shown that GA does indeed suffer nucleophilic attack by glutathione at C-19 without enzymatic activation as discussed below.^84^ In our own work, also discussed below, we hypothesized that the steric bulk of a substituent at C-19 might also cause a conformational change in the GA macrocycle arising from amide *trans* to *cis* isomerism as a result of steric strain.^85^

### 3.1 Natural Products

**Figure 4:**
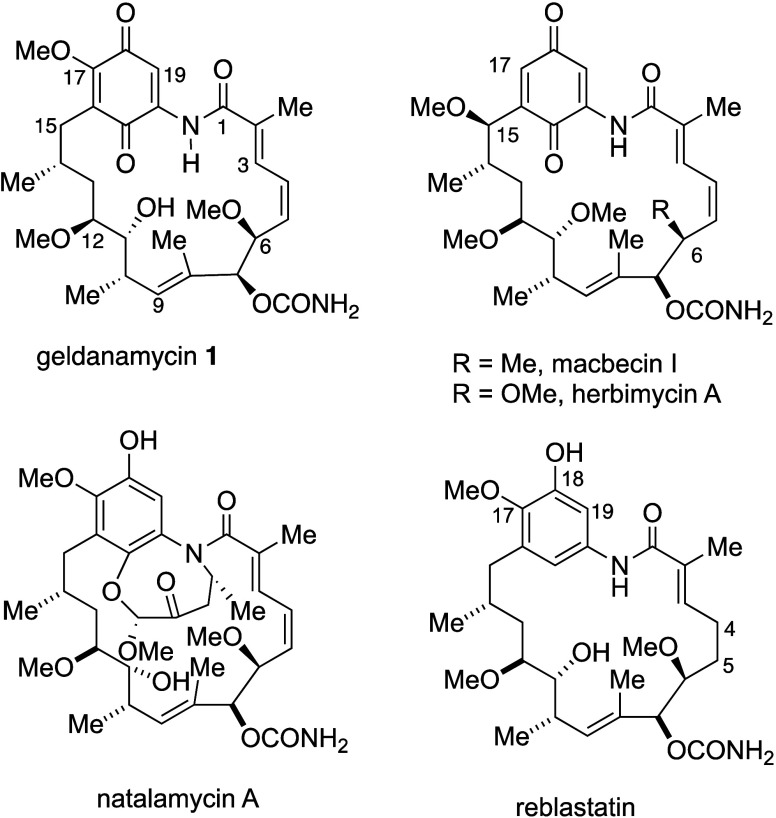
Comparison of structures of geldanamycin macbecins, herbimycins, natalamycin and reblastatin.

In contrast to naturally occurring geldanamycin derivatives that incorporate a substituent other than methoxy at C-17, where only 17-hydroxy,^86,87^ 17-amino,^39^ and 17-methyl^81^ derivatives have been described, a number of natural geldanamycins substituted at C-19 have been isolated from various sources. Also known are the closely related benzoquinone ansamycin natural products macbecins and herbimycins, both of which lack the C-17 methoxy group and have a slightly modified ansa-chain (Figure 4).^88,89^ More recently a number of nonquinone ansamycin natural products have been isolated and reported, including the natalamycins,^39^ and reblastatins (Figure 4) that have a modified ansa-chain with the 4,5-alkene reduced,^90,91^ and a number of other atypical benzenoid ansamycins and their congeners.^92^

Advances in mutasynthesis have led to a range of nonbenzoquinone analogues of GA employing different aromatic starter units for polyketide synthases.^93−95^ These studies have provided a range of novel benzenoid ansamycins, analogues of reblastatin, for biological evaluation. For example, the 17,18-unsubstituted, 19-fluoro analogue of reblastatin showed superior *in vitro* antiproliferative activity in ovarian (SKOV-3, 54 nM) and breast (MCF7, 18 nM) cancer cell lines compared to both 17-AAG (SKOV-3, 240 nM; MCF7 58 nM) and 17-DMAG (SKOV-3, 122 nM; MCF7 71 nM).^92,93^

C-19-substituted geldanamycin-based natural products include 19-sulfur, -oxygen and -carbon derivatives, albeit largely without discussion on the potential biosynthesis (with the exception of 19-(4-hydroxy-1′-methoxy-2′-oxopentyl) **13**), with the potential therefore for either a modified polyketide biosynthetic pathway to that known for GA, or a conjugate addition step on GA itself under the physiological conditions with various nucleophiles.

**Figure 5:**
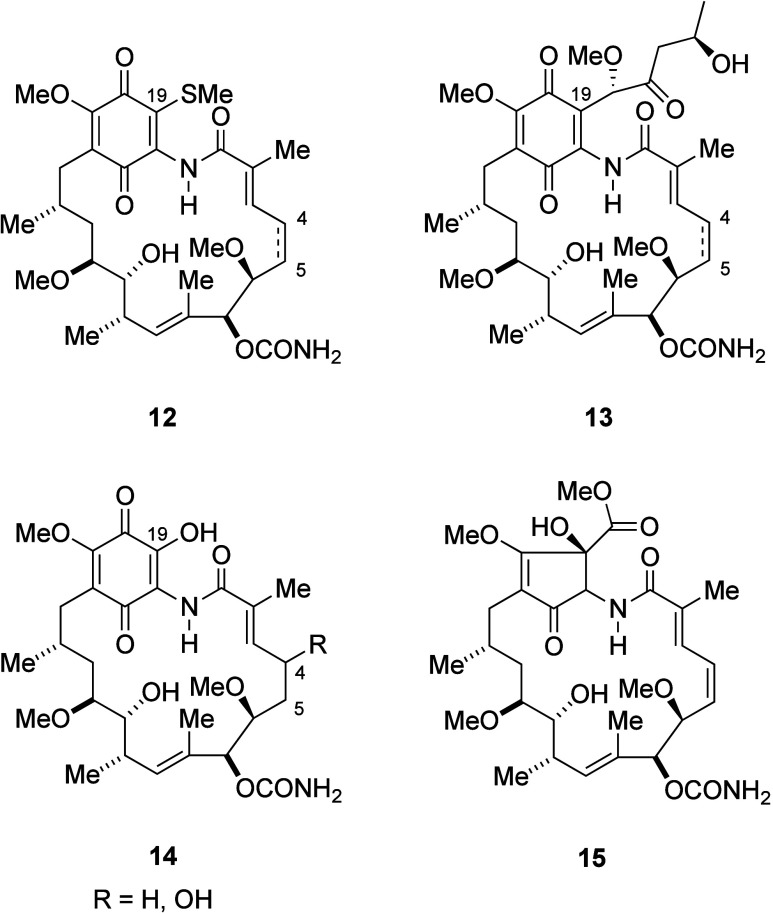
19-Substituted geldanamycin natural products and related ring-contracted cyclopentenone compound.

**Figure 6:**
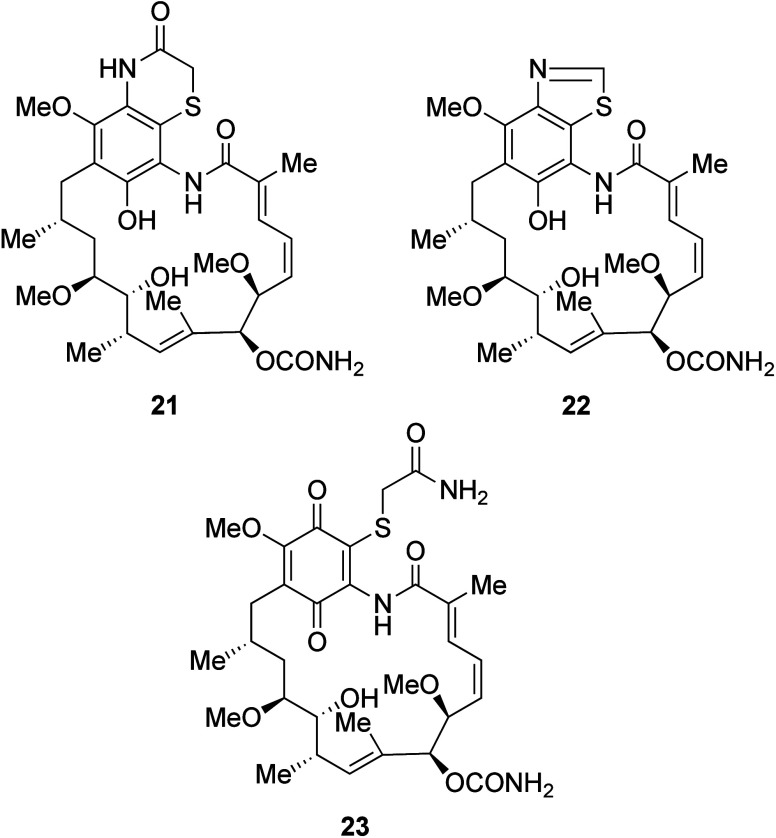
19-Thiazino, thiazolo and thioacetamido adducts of geldanamycin.

For C-19 sulfur-based derivatives, several publications have reported the isolation and characterization of 19-SMe geldanamycin **12** with or without the saturated chain at positions 4–5 (Figure 5).^39,86,87^ The antiproliferation activity has been tested against a panel of four cancer cell lines, but only decreased activity was observed vs. C-19 unsubstituted geldanamycins.^96,97^ However, the compounds did reduce the off-target toxicity to healthy cells (an issue in the clinic for many GA derivatives as discussed in Section 2.1), a phenomenon also observed with semisynthetic 19-substituted geldanamycins that have been shown to shut down a toxicity pathway with the conjugation of biological nucleophiles at C-19 (more details below).^84,85,98^ Also reported are the thiazino **21**, thiazolo **22** and thioacetamido **23** adducts (Figure 6), further described below since these appear to be more a result of bioengineering than strictly natural products.^99^

For carbon-substituted C-19 natural geldanamycins, two publications in quick succession described the isolation of the 4-hydroxy-1′-methoxy-2′-oxopentyl derivative **13** from two strains of *Streptomyces*.^39,96^ Interestingly, the compound was found to be significantly more water-soluble than GA itself (15400 μg/mL and 7705 μg/mL for the 4,5-unsaturated and 4,5-saturated analogues, respectively vs. 2–7 μg/mL for GA), likely a combination of the extra hydrophilic functionalities coupled with a conformational switch observed on 19-substitution as discussed further below.^85^ As with other C-19-substituted derivatives, the compounds also exhibited much lower toxicity to healthy liver cells (IC_50_ = 14.6 μM and 22.4 μM for the 4,5-unsaturated and 4,5-saturated analogues, respectively vs. 0.3 μM for GA). Poulsen and Clardy proposed a biosynthesis for **13** from geldanamycin hydroquinone. They suggested that the 19–C-C bond is formed through a link to a speculative electrophilic component such as (*E*)-2-oxopent-3-enal. However, it is debatable whether this is more likely to proceed via the *hydroquinone* through electrophilic aromatic substitution-type chemistry or via the *quinone* using the enamide functionality to react with such electrophiles. 19-Methylgeldanamycin **33** (R = Me) (further discussed below) has also been reported as a metabolite from *Streptomyces* sp. 11–1–2, detected by LC-MS and compared with ion identity molecular networking, albeit with little further discussion and no further analysis/biological testing reported in the article.^100^

C-19 hydroxygeldanamycin **14** has been reported with either saturation at positions 4–5 or with a further hydroxyl group at position 4, and this can give rise to a related cyclopentenone natural product **15** that is noteworthy.^86^ Isolated initially from a microbe found in an abandoned Kentucky coal mine, it appears to be the result of a benzylic acid-type ring-contraction from 19-hydroxygeldanamycin. This represented a new type of potential Hsp90 inhibitors (dubbed the mccrearamycins) and has since been discovered in an extract from *Streptomyces malaysiensis* and tested for potential activity against various cancer cell lines, albeit with diminished activity relative to the parent GA derivatives observed in all cases.^87^

### 3.2 N-Substituted Analogues

**Scheme 5:**
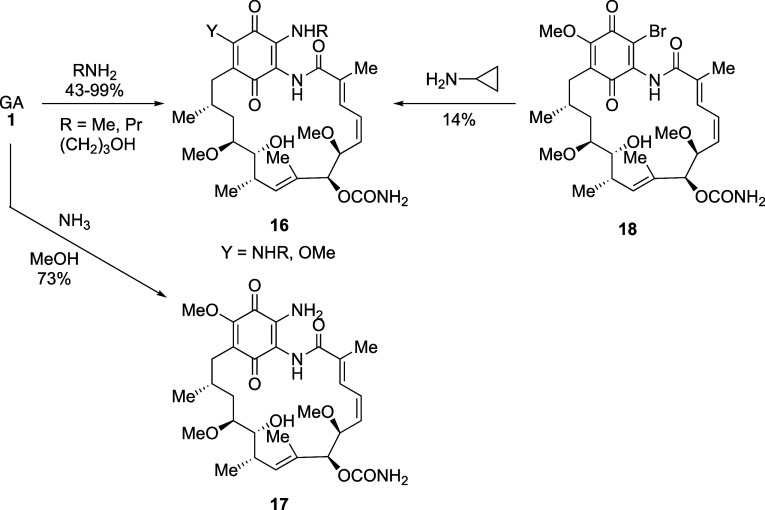
19-Amino Geldanamycin Derivatives with/without Concurrent 17-Amino Substitution

While substitution at C-17 with nitrogen nucleophiles has received considerable attention, C-19 nitrogen derivatives are less well-known despite the fact that they are readily available. They can be accessed through either conjugate addition–oxidation or addition–elimination processes, and are reported to occur with hindered amines or under forcing conditions (with or without concomitant C-17 amination). Thus, a range of 17,19-dinitrogen-substituted geldanamycins **16** and 19-aminogeldanamycin **17** are accessible from GA **1** following exposure to the corresponding amine (Scheme 5).^35^ The same class of compound can be accessed from the corresponding 19-halo derivative (*e.g*. 19-bromogeldanamycin **18**), although in modest reported yield (Scheme 5).^36^ Such adducts have been found to exhibit both *in vitro* and *in vivo* antiviral activity.^101,102^ albeit with *in vitro* HER2 inhibition assays in SkBr-3 cells the 17,19-diamino adducts were less potent than the mono C-17 adducts.^36^ With diamines such as *o*-phenylenediamine, the adduct is hypothetically possible at C-17 or C-19 along with condensation at the C-18 quinone carbonyl group. However, Rinehart and co-workers reported the near exclusive C-17 pathway, giving the phenazine analogues **8** (Scheme 4).^12^

### 3.3 S-Substituted Analogues

**Scheme 6:**
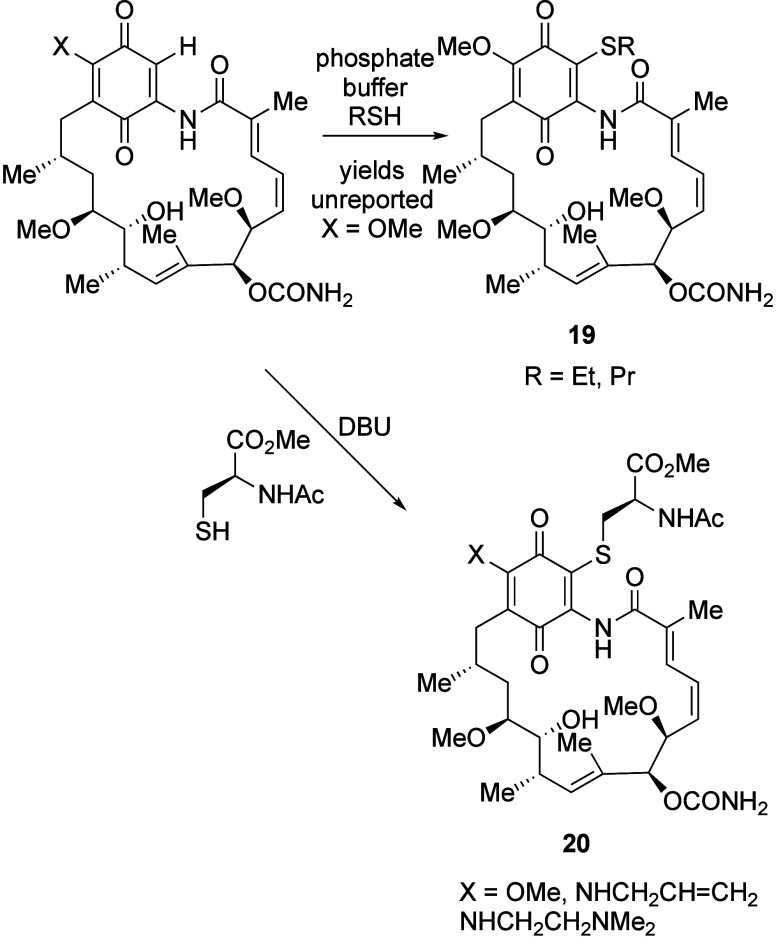
19-Thio-Adducts from the Conjugate Addition of Sulfur Nucleophiles, Including the Glutathione Mimic *N*-Acetylcysteine Methyl Ester

In addition to the natural products discussed above, there have been numerous synthetic examples of 19-sulfur-substituted GA derivatives **19** and **20** reported. This can be achieved directly from GA itself through a conjugate addition mechanism with thiols^103−105^ or thiolate salts^85^ in moderate yield (where reported) (Scheme 6). Indeed, this has been proposed and investigated as a pathway leading to the toxicity of such benzoquinone ansamycins; specifically the conjugation of thiol-containing biological nucleophiles such as glutathione in the liver, the adducts of which have indeed been observed and reported.^84,106^

**Scheme 7:**
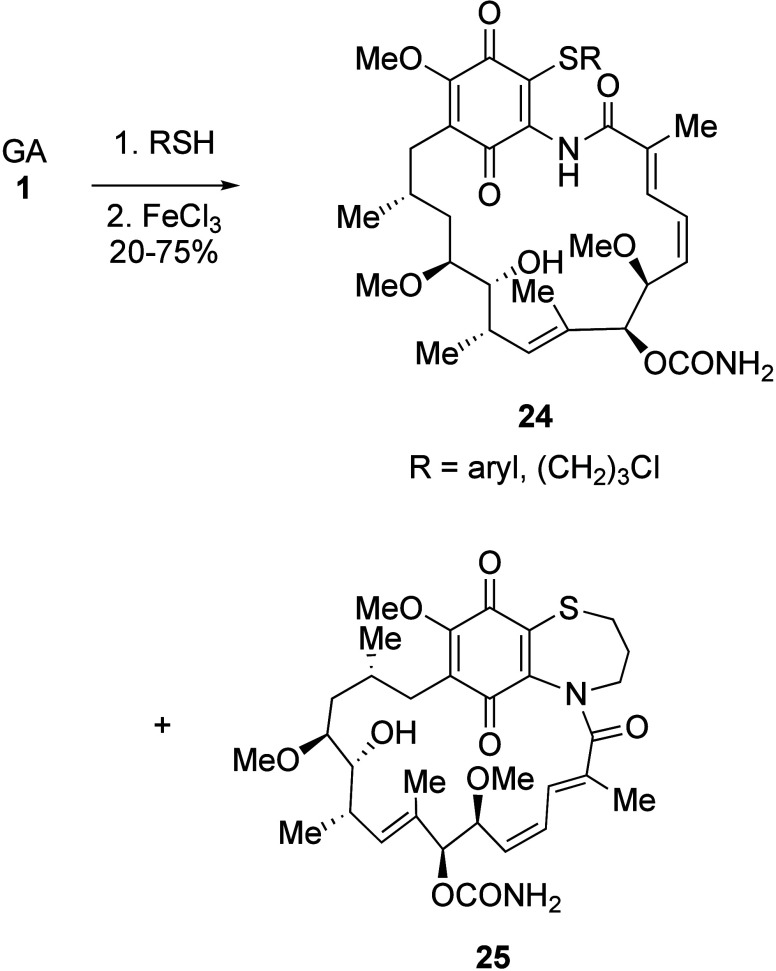
19-Thio-Adducts from the Conjugate Addition of Sulfur Nucleophiles, Including 3-Chloropropylthiol

17,19-Bis thio-adducts have also been reported in the literature, resulting from exposing *S*. *hygroscopicus* N02Z-0421 to an enhanced s-methionine feedstock during culturing, isolated along with the 19-mono thiomethyl adduct of GA. A biosynthesis/semisynthesis combination approach was employed for the synthesis of a series of 19-substituted geldanamycin and related derivatives (Figure 6, Scheme 7). Standard fermentation for GA along with cysteine or aminoethanethiol feedstocks at a range of pHs allowed access to compounds **21**–**23** in moderate yield (Figure 6). Furthermore, exposure of GA to a range of aryl or alkyl thiols, with subsequent oxidation, where required, gave the 19-substituted derivatives **24** in low-excellent yield. Interestingly, several of the adducts were found to have more stable hydroquinone forms than the parent GA derivatives and, as such, FeCl_3_-promoted oxidation was required to access the quinone series (Scheme 7).^99^ Also isolated was the annulation product resulting from cyclization of the alkyl chloride to the position-1 nitrogen **25** (Scheme 7).^99^ All compounds exhibited superior water solubility vs. GA itself, possibly due to the conformational and polarity changes discussed below, but also lower bioactivity compared to the parent compound against human liver cancer (HepG2) cells.^99^

### 3.4 O-Substituted Analogues

**Scheme 8:**
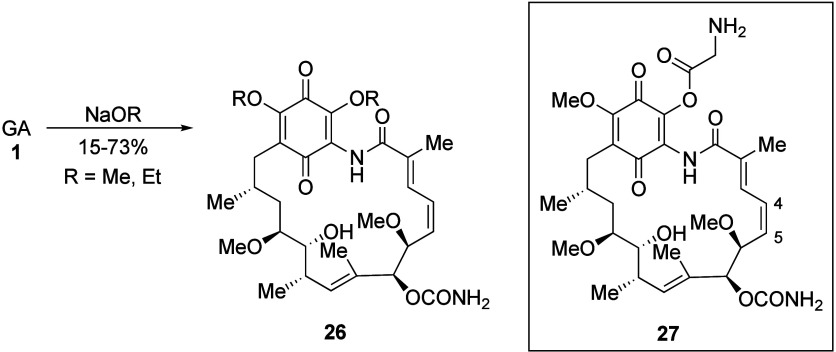
19-Alkoxy and 17,19-Dialkoxy Adducts **26** from the Conjugate Addition of Oxygen Nucleophiles, and the Naturally Occurring Glycino Derivative **27**

As with the 17-position, oxygen substituents at C-19 can be introduced through a conjugate addition and subsequent oxidation procedure (Scheme 8). Thus, the synthesis of 17,19-dialkoxy geldanamycin derivatives **26** through treatment of GA **1** with an excess of the corresponding alkoxide has been described.^35^ The OEt 17,19-bis adduct (**26**, R = Et) was found to be particularly toxic to P19-derived neurons (0% cell viability), while the corresponding methoxy derivative (**26**, R = Me) was actually reported to possess a wide therapeutic index between neuroprotective and neurotoxic activities, which the authors felt gave the species potential for further development for neurodegenerative therapy.^35^ Furthermore, the 19-glycine derivative **27** was obtained through a biosynthetic procedure, as reported in a 2010 Chinese patent.^107^ The 4,5-dihydro analogue of the 19-glycine derivative **27** has also been isolated, and is less toxic than GA toward healthy HepG2 cells (302 μM vs. 0.59 μM) together with better aqueous solubility.^108^

### 3.5 C-Substituted Analogues

**Scheme 9:**
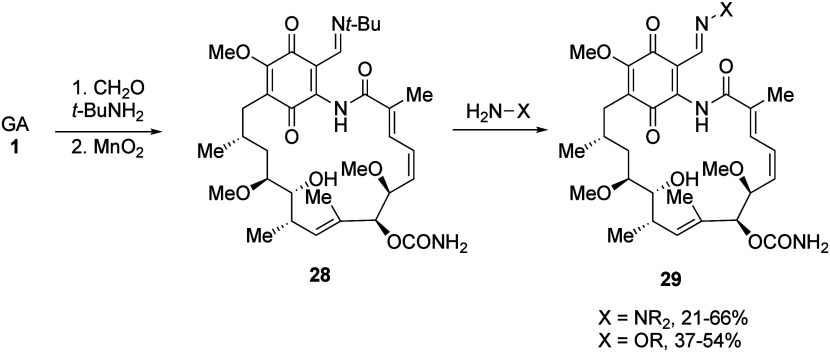
Mannich-Oxidation to C-19 Geldanamycin Adducts and Subsequent Substitution Products with Hydrazines and Hydroxylamines

Carbon-based substitution at C-19 has been achieved directly from GA itself. Thus, a series of formyl hydrazones **29** (X = NR_2_) and oximes **29** (X = OR) have been reported, resulting from a Mannich-oxidation sequence followed by substitution with a variety of hydrazines and hydroxylamines in modest to good yield (Scheme 9),^11,109^ although the hydrolysis to the corresponding aldehyde was not possible to achieve.^11^ The adducts were tested *in vitro* for inhibition of reverse transcriptase (RT) derived from Rauscher leukemia virus along with cytotoxicity against BALB 3T3 cells, with moderately good activity observed, especially for the *O*-benzyloxime (**29**, X = OCH_2_Ph) with 81% RT inhibition reported at a concentration of 0.025 μmol mL^–1^ (GA was inactive in this assay) along with up to 500 times lower toxicity than GA.^11^ Interestingly, this was also reported alongside a considerable reduction in cytotoxicity compared to GA, especially for the hydrazone derivatives.^11^ This is a phenomenon we have also observed for C-19 adducts, with further investigations and explanations described below.

More recently, screening of a library of over 1900 small molecules identified the morpholine hydrazone (NSC255112) **29** (X = 1-morpholino) as a potent inhibitor of not only *O*-glycosylation, but also other Golgi-localized glycosylation processes, including elaboration of *N*-glycosylation and biosynthesis of glycosaminoglycans, notably without substantially affecting secretion of glycoproteins.^110^

**Scheme 10:**
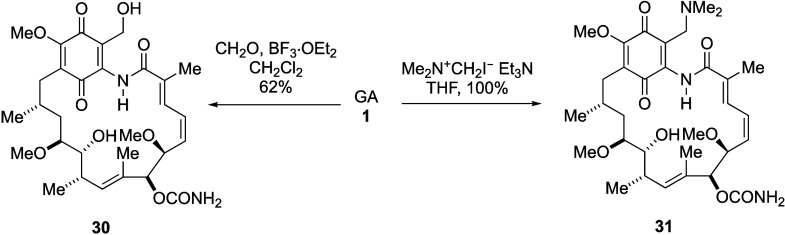
19-Hydroxymethyl and Aminomethyl Geldanamycin Derivatives from Mannich- or Aldol-like Procedures

In a similar approach, we have accessed the lower oxidation level C-19 adducts based on similar reactions with formaldehyde or Eschenmoser’s salt to give compounds **30** and **31**, respectively in good-to-excellent yield (Scheme 10).^111^

### 3.6 Carbon Substituents at C-19 Reduce Toxicity and Induce Conformational Change in the BQA Macrocycle

**Scheme 11:**
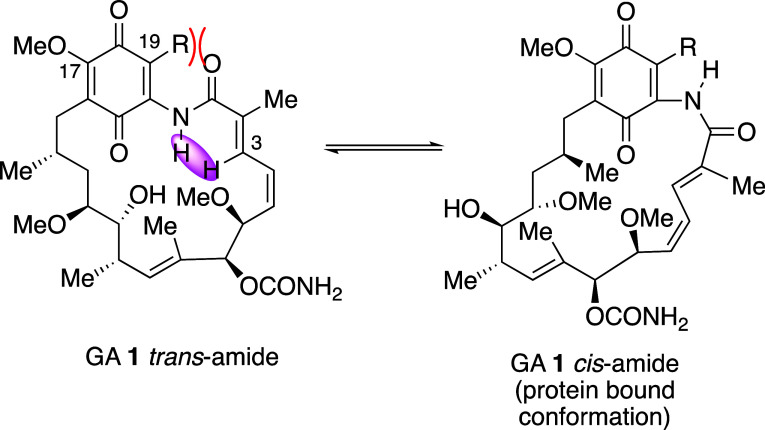
*trans*–*cis* Amide Isomerization in Geldanamycin BQAs, Showing the Possible Effect of a Substituent at C-19 Favoring the *cis*-form

As already noted, the use of GA itself is limited due to hepatotoxicity,^34^ possibly as a result of reaction with biological nucleophiles such as glutathione at the reactive 19-position of the quinone ring.^84,106,112^ Therefore, we reasoned that the conjugation of glutathione (or other nucleophiles) might be inhibited by blocking the 19-position with a suitable substituent, with a consequent reduction of toxicity. We also reasoned that a conformational change in the macrocyclic ring might occur due to amide *trans* to *cis* isomerism as result of the steric bulk of a substituent at C-19 (Scheme 11). In the solid state, the BQA macrocyclic lactams are known to adopt an extended *trans*-amide conformation as shown by X-ray crystal structures of GA **1** itself,^113^ and 17-azetidinyl-17-demethoxygeldanamycin.^114^ NMR spectroscopic studies on the solution conformation of the 17-azetidinyl derivative also suggested a *trans*-amide conformation as evidenced by a strong nuclear Overhauser effect (NOE) enhancement between the NH and the alkene H-3 as indicated in Scheme 11.^114^ However, on binding to Hsp90 (yeast or human), both GA **1** and 17-DMAG **3** adopt a more closed conformation with a *cis*-amide bond as shown by protein crystallography,^66,67,115^ although the equilibrium (Scheme 11) between *trans*- and *cis*-forms is a matter of some debate.^116,117^

More recent X-ray crystallography studies have shown that C-17 amino compounds derived from both 4-amino-1-benzylpiperidine and 4-amino-1-ethoxycarbonylpiperidine appear to adopt a conformation in the solid state that is halfway between the *trans*-amide of “free” geldanamycins and the *cis*-amide of protein-bound derivatives.^75^ Such intermediate conformations may be relevant to the interconversion of *trans*- and *cis*-amide conformers.

**Scheme 12:**
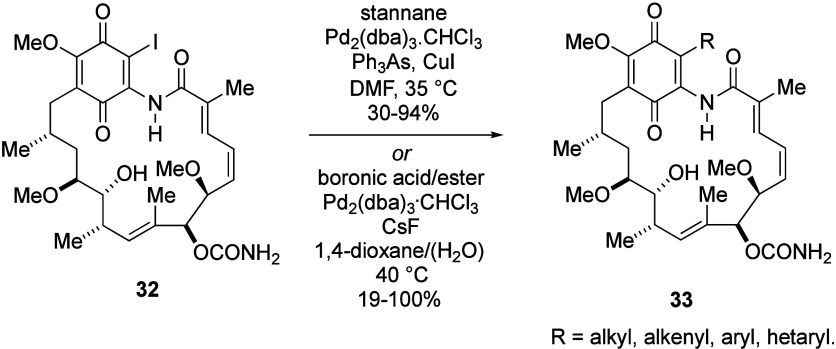
Synthesis of 19-Substituted Geldanamycin Derivatives by Stille or Suzuki–Miyaura Coupling Reactions

In order to investigate the effect of a substituent at C-19 on the conformation and toxicity of GA derivatives, we set out to synthesize a wide range of stable analogues, which we refer to as 19-BQAs, with a diverse set of substituents at the 19-position.^85^ We have reported several approaches to C-19 carbon substitution of GA accessed via 19-iodogeldanamycin **32** through metal-catalyzed/mediated coupling processes. Our initial focus involved a specialized Stille-coupling approach, employing triphenylarsine and copper iodide and allowed access to a wide range of 19-substituted derivatives **33** in modest to excellent yield (Scheme 12).^85,111^

**Scheme 13:**
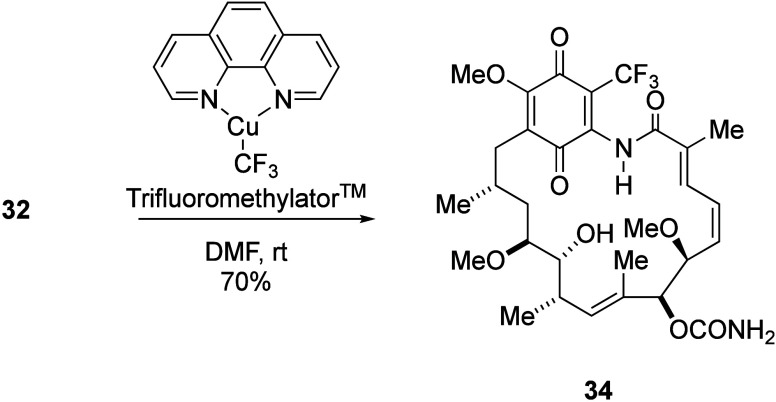
19-Trifluoromethylation through a Copper-Mediated Coupling

Although purification allowed tolerable levels of trace metals in the coupled products (10.5 ppm Pd, 7.9 ppm As, 7.9 ppm of Sn and undetectable Cu following the coupling and 2.0 ppm Pd, 2.5 ppm As, 0.5 ppm of Sn and 4.9 ppm of Cu following the amino-substitution at C-17), we subsequently developed a more “benign” Suzuki–Miyaura coupling protocol, taking inspiration from the successful coupling reactions of the C-17 triflate derivative of GA in work previously described in Scheme 3.^80^ This gave the same diverse array of 19-BQAs in generally excellent yield, and also allowed access to compounds we were unable to prepare through the Stille protocol.^111,118^ We have also described a copper-mediated approach for the introduction of a trifluoromethyl group at position 19 (compound **33**) in good yield, employing the trifluoromethylator reagent developed by Hartwig (Scheme 13).^111,119^ Furthermore, nonmetal promoted methods for derivatizing C-19 such as shown in Schemes 9 and 10 could prove more “benign” and therefore attractive to industry, but with reduced substrate scope compared to the coupling procedures (*e.g*. C–C aryl derivatives would be difficult to access).

**Figure 7:**
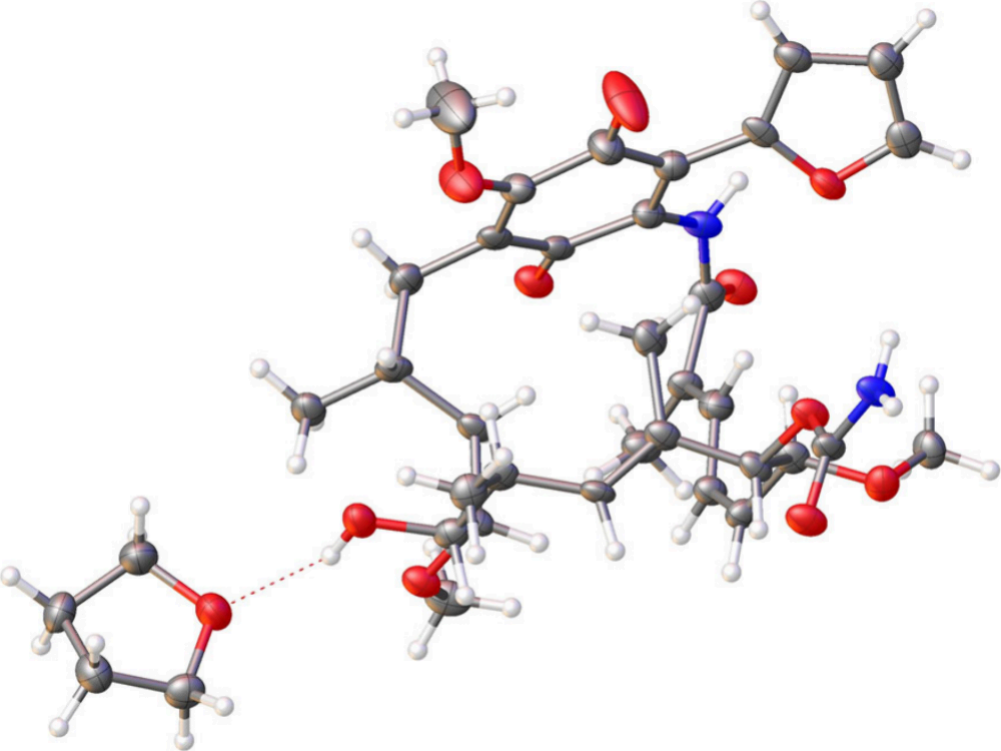
X-ray crystal structure of 19-(2-furyl)geldanamycin (**33**, R = 2-furyl) (the compound crystallizes with one molecule of THF).^85^

With a range of 19-substituted geldanamycins in hand, we were able to investigate whether the proposed conformational switch caused by amide *trans*–*cis* isomerization (Scheme 11) was borne out by experiment. It was observed that all the 19-substituted derivatives exhibited ^1^H chemical shift patterns in their NMR spectra that were significantly different from GA itself, suggesting a change in the environment of many of the protons, influenced by a potential conformational change of the amide. Further NMR studies, together with molecular modeling, strongly suggested that in solution, the 19-substituted geldanamycins predominantly adopted a more closed, *cis*-amide conformation.^85^ Evidence from X-ray crystallographic studies of 19-(2-furyl geldanamycin (**33**, R = 2-furyl) showed that the molecule clearly adopted a closed *cis*-amide conformation in the solid state (Figure 7),^85^ in direct contrast to previous studies of 19-unsubstituted geldanamycins that adopt the *trans*-amide conformation.^113,114^

As described above, we had also reasoned that the conjugation of nucleophiles might be inhibited by blocking the 19-position, and this indeed proved to be the case. Whereas 19-unsubstituted geldanamycins reacted readily with the glutathione mimic *N*-acetylcysteine methyl ester at C-19 (Scheme 6), the corresponding 19-methyl- and -phenyl derivatives showed no reaction with the thiol.

**Figure 8:**
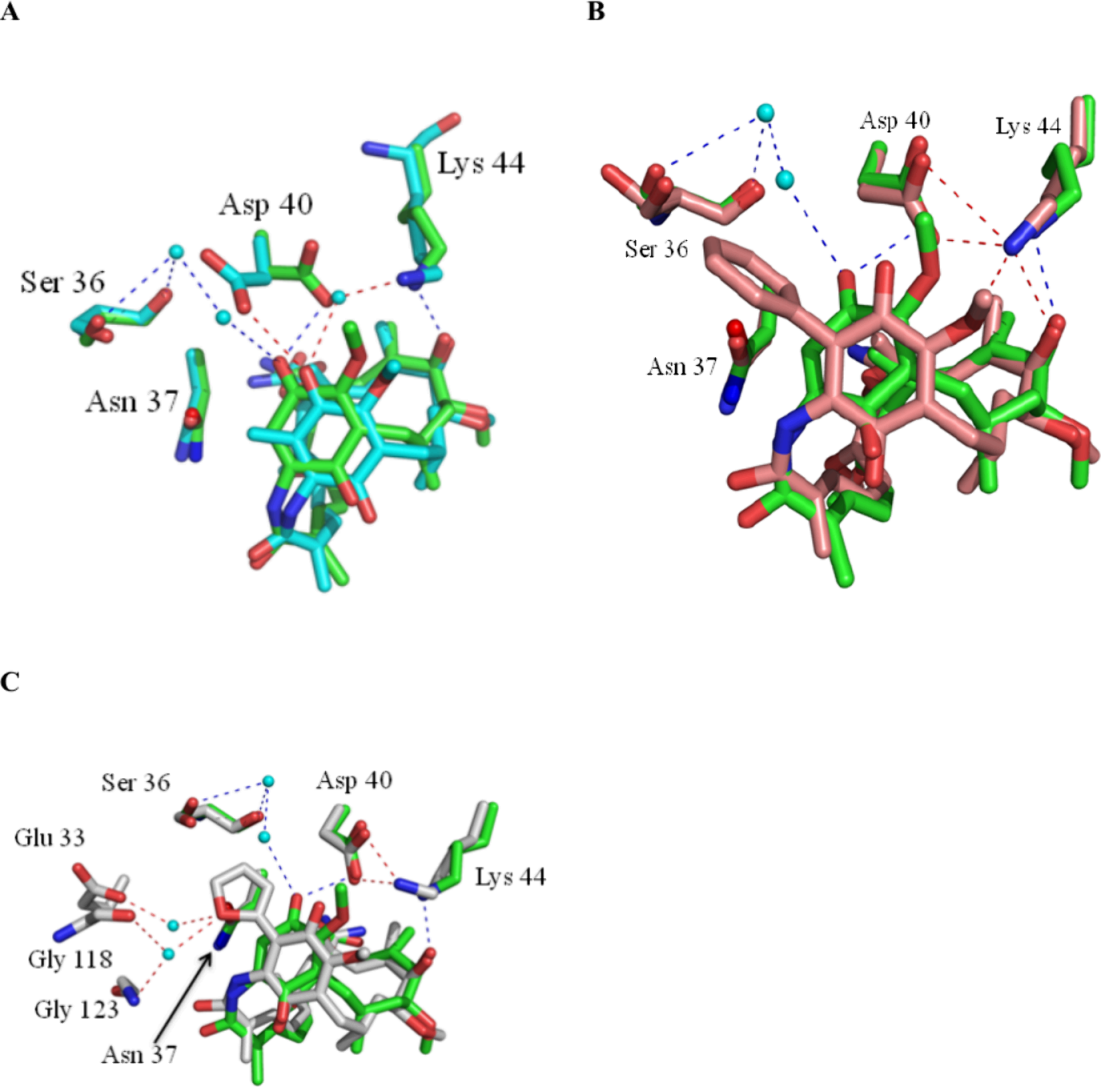
Comparison of the binding of GA with 19-substituted analogues **33** to the *N*-terminal domain of yeast Hsp90, as determined by protein X-ray crystallography showing the similarity of the bound conformations and interactions with Hsp90 residues; **A**, GA **1** (green) and 19-methyl geldanamycin **33** (R = Me) (cyan) with Hsp90 (green and cyan residues, respectively; **B**, GA **1** (green) and 19-phenyl geldanamycin **33** (R = Ph) (salmon) with Hsp90 (green and salmon residues, respectively) ; **C**, GA **1** (green) and 19-(2-furyl) geldanamycin **33** (R = 2-furyl) (gray) with Hsp90 (green and gray residues, respectively). Reprinted with permission from ref (85). Copyright 2013 Springer Nature Limited.

The hypothesis that 19-substituted BQAs did not react with thiols, were less toxic in cellular systems yet still maintained Hsp90 inhibitory and growth inhibitory activity in cancer cells was further validated in a series of experiments using 19-phenyl and 19-methyl geldanamycin derivatives **33** (R = Me, Ph) primarily in the DMAG series to provide proof of principle.^85,98^ Isothermal calorimetry and X-ray crystallography (Figure 8) clearly established that 19-Me-DMAG bound to the yeast *N*-terminal domain of Hsp90 although with diminished affinity relative to GA (K_d_ = 16.3 μM vs. 2.9 μM for GA), likely due to a shift in the position of the quinone, as is shown in Figure 8 for various C-19 substituents vs. GA. Parent BQAs (GA, 17-AAG and 17-DMAG) reacted directly with thiol groups including the biologically relevant thiol, glutathione, while their 19-substituted derivatives were devoid of thiol depleting activity. 19-Phenyl and 19-methyl DMAG were validated as Hsp90 inhibitors in human breast cancer and human neuroblastoma cell lines using the molecular signature of depletion of Hsp90 client proteins and the induction of alternative heat shock protein chaperones, Hsp70 in breast cancer cells and both Hsp70 and Hsp27 in neuroblastoma cells.

The growth inhibitory potential of 19-substituted BQAs was examined using the NQO1 overexpressing cell line MDA468/NQ16. Geldanamycin (60-fold), 17-AAG (45-fold) and 17-DMAG (17-fold) were more active than their 19-phenyl analogues.^98^ In addition, 19-phenyl analogs were between 2 to 6-fold more potent in growth inhibition studies in NQO1 overexpressing cells (MDA468/NQ16) relative to the NQO1 null isogenic cell line (MDA468) suggesting, that similar to 19-unsubstituted parent compounds, 19-substituted BQAs are more active in the presence of NQO1.^98^ Using human breast cancer cell lines MDA468 and BT474, biomarkers of Hsp90 inhibition (Raf-1, Akt, HER2 degradation and Hsp70 induction) were examined at a single concentration (5 μM) and 19-methyl and 19-phenyl 17-DMAG demonstrated near equal potency compared to 17-DMAG.

A central question was whether 19-substituted analogues exhibited decreased toxicity to normal cells and particularly to liver cells since hepatotoxicity has often been associated with use of the BQA series of Hsp90 inhibitors. Both 19-phenyl and 19-methyl 17-DMAG showed markedly decreased toxicity to freshly isolated mouse hepatocytes and a mouse hepatocyte cell line in culture.^98^ In studies in freshly isolated mouse hepatocytes, 17-DMAG demonstrated significantly lower survival and higher aspartate aminotransferase release when compared to 19-methyl and 19-phenyl 17-DMAG. In LC_50_ studies using the TAMH cell line 17-DMAG was 203-fold more cytotoxic than 19-methyl 17-DMAG and 66-fold more cytotoxic than 19-phenyl 17-DMAG. In studies to measure redox cycling in isolated mouse and human liver microsomes 19-methyl 17-DMAG stimulated markedly less oxygen consumption compared to 17-DMAG.^98^

In summary, 19-substituted BQAs did not react with thiols, demonstrated lower rates of redox cycling and were markedly less toxic to normal human cells and to mouse hepatocytes when compared to the parent compounds. In cancer cells, 19-phenyl-substituted analogues were less active compared to unsubstituted BQAs and similar to the parent BQAs 19-phenyl analogues were more active in the presence of NQO1 suggesting a role for intracellular reduction to the hydroquinone in promoting growth inhibition.

In a departure from the oncology arena, in collaboration we also examined the effect of our novel 19-BQAs in HIV.^28^ Hsp90 is required for HIV-1 gene expression,^120^ and for enhanced HIV-1 replication in conditions of hyperthermia (fever),^121^ and controls HIV-1 reactivation from latency by modulating the NF-κB pathway.^122^ Hence BQA Hsp90 inhibitors can suppress HIV-1 reactivation from latency. Unfortunately in all three series of BQAs (geldanamycin, 17-AAG and 17-DMAG) incorporation of a methyl or phenyl substituent at C-19 reduced their potency by more than 10-fold.^122^

It was mentioned earlier (Sections 2.2 and 3.4) that certain 17- and 19-alkoxy derivatives of GA were neuroprotective,^35^ and the involvement of Hsp90 in neurological disorders has been widely investigated of late.^123^ Some Hsp90 client proteins are believed to play an important role in protein aggregation underlying neurodegenerative diseases,^20−22^ examples of which include α-synuclein, tau, and Huntingtin proteins. Many other chaperones, kinases and transcription factors are Hsp90 clients.

BQA Hsp90 inhibitors may be beneficial in protection against neurodegenerative diseases via a number of mechanisms. These include a reduction in the load of potentially damaging Hsp90 client proteins, induction of the heat shock response via HSF-1 leading to activation of other heat shock proteins and proteasomal degradation, and activation of protective autophagy or other mechanisms involved in protein homeostasis.^22,25,124−126^ α-Synuclein and its mutant forms have been implicated in the etiology of Parkinson’s disease and Hsp90 inhibitors have been shown to inhibit α-synuclein aggregation and toxicity in multiple model systems.^20,127,128^ In agreement with the observed protective effects of Hsp90 inhibitors against α-synuclein toxicity, we found that 19-phenyl geldanamycin could inhibit the biochemical and toxic effects induced by overexpression of mutant A53T α-synuclein in human dopaminergic SH-SY5Y cells.^129^ A53T mutant α-synuclein protein stimulated formation of α-synuclein oligomers, inhibited ubiquitin-dependent proteasomal activity and induced cellular toxicity, all of which could be ameliorated by 19-phenyl geldanamycin **33** (R = Ph). Induction of a marked heat shock response and activation of autophagy observed with 19-phenyl geldanamycin **33** (R = Ph) probably contributed to cellular protection against A53T mutant α-synuclein and inhibition of mTOR/p70S6K signaling may also have played a role.^129^

The Hsp90 network is extremely complex including the involvement of Hsp90 homo and hetero oligomers, diverse client proteins, multiple cochaperones and post-translational modifications functioning in a context-dependent manner.^17,130−132^ Such complexity provides many opportunities for selective drug design and targeting. For example, in both *in vitro* and *in vivo* model systems, an Hsp90 cochaperone Aha-1 was shown to drive the formation of oligomers and insoluble forms of tau protein thought to play an important role in Alzheimer’s disease, while targeting the interaction of Hsp90 and Aha-1 with a small molecule inhibited tau aggregation.^133^ Inhibition of the Hsp90/Cdc37 cochaperone complex has also been of considerable interest for drug design efforts.^134,135^ Another attractive approach is to activate specific features of the Hsp response which may allow targeting of neuroprotective responses in a more focused manner.^136^

## 4 Conclusion

This Perspective has highlighted the role played by the naturally occurring benzoquinone ansamycin geldanamycin in drug discovery, particularly in the development of Hsp90 inhibitors for applications in oncology. Despite GA providing an excellent lead compound, it was not progressed to the clinic, due to unacceptable liver toxicity. Nevertheless, a large number of semisynthetic compounds formed by nucleophilic addition–elimination of amines at C-17 have been widely investigated. However, despite extensive studies, with three such compounds entering clinical trials, a successful BQA drug has remained elusive.

As to the future, given the rich chemistry of the 19-position in GA, and the biological properties of the resulting compounds discussed above, we believe that such analogues merit more attention from medicinal chemists. The compounds are readily prepared by metal-catalyzed coupling reactions of 19-iodogeldanamycin, a reaction that could in principle be extended to coupling of O-, N- and S-groups. In the 19-carbon-substituted series, the compounds are markedly less toxic to normal human cells than GA itself, while retaining Hsp90 inhibitory activity, suggesting possible applications in other arenas such as neurodegenerative disease. Also, following promising initial results with BQAs in NRF2-activated cancers, C-19 quinone-modified geldanamycin derivatives should be investigated for such applications to determine their efficacy coupled with lower off-target toxicity.

It also seems that the hydroquinone analogues of the various geldanamycins remain relatively unexplored given that reduction of the quinone moiety can lead to increased Hsp90 inhibition by increased H-bonding and more favorable binding in the ATP binding site of Hsp90. If hydroquinones are thought to be too susceptible to reoxidation, one could envisage derivatives where the phenols are protected with groups that could be manipulated for ease (or not) of removal.

Finally, the complexity of the Hsp90 chaperone system allows considerable scope for new drug design and targeting in multiple disease states. Innovative efforts to design isoform-specific inhibitors of Hsp90 to potentially avoid toxicities observed with pan-Hsp90 inhibitors, targeting of Hsp90-co-chaperone complexes and activation of specific aspects of the heat shock response are all active areas of research and have been informed by previous studies using the geldanamycin class of Hsp90 inhibitors.

## Abbreviations Used

17-AAG: 17-allylamino-17-demethoxygeldanamycin (tanespimycin)
17-AG: 17-amino-17-demethoxygeldanamycin
17-DMAG: 17-*N*,*N*-dimethylaminoethylamino-17-demethoxygeldanamycin (alvespimycin)
19-BQA: 19-substituted benzoquinone ansamycin
ATP: adenosine triophosphate
BQA: benzoquinone ansamycin
Cdc37: cell division cycle 37, an Hsp90 cochaperone
CoA: coenzyme A
DMF: *N,N*-dimethylformamide
GA: geldanamycin
GRP94: glucose-regulated protein 94
HER2: human epidermal growth factor receptor 2
HSF-1: heat shock factor 1
Hsp70: heat shock protein 70
Hsp90: heat shock protein 90
mTOR: mammalian target of rapamycin
NMR: nuclear magnetic resonance (spectroscopy)
NOE: nuclear Overhauser effect
NQO1: NAD(P)H:Quinone oxidoreductase 1
NRF2: nuclear factor erythroid 2-related factor 2
RDDP: RNA-dependent DNA polymerase
RLV: Rauscher leukemia virus
Tf: trifluoromethylsulfonyl
TNF: tumor necrosis factor
TRAP-1: TNF receptor associated protein 1
